# Biocatalytic synthesis of 2-fluoro-3-hydroxypropionic acid

**DOI:** 10.3389/fbioe.2022.969012

**Published:** 2022-08-17

**Authors:** Wei Liu, Shan Yuan, Miaomiao Jin, Mo Xian

**Affiliations:** ^1^ CAS Key Laboratory of Biobased Materials, Qingdao Institute of Bioenergy and Bioprocess Technology, Chinese Academy of Sciences, Shandong, China; ^2^ University of Chinese Academy of Sciences, Beijing, China

**Keywords:** 2-fluoro-3-hydroxypropionic acid, fluoride, one-pot synthesis, whole-cell transformation, biopolymer

## Abstract

Fluorine has become an important element for the design of synthetic molecules for use in medicine, agriculture, and materials. The introduction of fluorine atoms into organic compound molecules can often give these compounds new functions and make them have better performance. Despite the many advantages provided by fluorine for tuning key molecular properties, it is rarely found in natural metabolism. We seek to expand the molecular space available for discovery through the development of new biosynthetic strategies that cross synthetic with natural compounds. Towards this goal, 2-fluoro-3-hydroxypropionic acid (2-F-3-HP) was first synthesized using *E. coli* coexpressing methylmalonyl CoA synthase (MatBrp), methylmalonyl CoA reductase (MCR) and malonate transmembrane protein (MadLM). The concentration of 2-F-3-HP reached 50.0 mg/L by whole-cell transformation after 24 h. 2-F-3-HP can be used as the substrate to synthesize other fluorides, such as poly (2-fluoro-3-hydroxypropionic acid) (FP3HP). Being entirely biocatalytic, our procedure provides considerable advantages in terms of environmental and safety impacts over reported chemical methods.

## Introduction

Organic fluorides are important compounds that are widely used in the fields of pharmaceuticals, molecular imaging, and materials (Klopries et al., 2014; Wu et al., 2020a; Cheng and Ma, 2021). Fluorinated natural products are extremely rare in nature (Cheng and Ma, 2021). However, the introduction of fluorine atoms into organic compound molecules can often give these compounds new functions and make them have better performance (Klopries et al., 2014; Ningning et al., 2020; Tu et al., 2020). The target compounds doped with fluorine are endowed with stronger stability and activity (Tu et al., 2020), longer half-life, and better bioabsorbability (Ningning et al., 2020), especially in the fields of pharmaceutical intermediates, cancer treatment (Lowe et al., 2019), antiviral agents, photovoltaics, diagnostic probes (Onega et al., 2010) and bioinspired materials (Ningning et al., 2020). For example, Benjamin W. Thuronyi et al. reported the introduction of fluorine into poly (3-hydroxybutyric acid) (P3HB) to obtain poly (2-fluoro-3-hydroxybutyric acid-co-3-hydroxybutyric acid) (poly (FHB-co-HB)), and the results show that the glass transition temperature of poly (FHB-co-HB) is much lower than that of P3HB (Thuronyi et al., 2017).

Despite the many advantages provided by fluorine for tuning key molecular properties, it is rarely found in natural metabolism, which limits the application of fluorine-containing compounds (Thuronyi and Chang, 2015; Cheng and Ma, 2021). Accordingly, the artificial synthesis of fluorinated compounds has become an important alternative in modern society. Nonetheless, these conventional chemical synthesis methods required precious metals, toxic and contaminating chemical reagents, high temperature and pressure and extreme conditions (Berger et al., 2020; Rodrigo et al., 2020). This is not conducive to the sustainable development of green chemicals and the global economy.

In contrast, the biosynthesis methods are good supplements in the field of chemical synthesis, especially for fluorinated compounds (Wu et al., 2020a). At present, of all enzyme-catalyzed synthesis methods, the direct formation of the C-F bond by fluorinase is the most effective and promising method (Cheng and Ma, 2021). Fluorinase can participate in biological metabolic pathways and synthesize valuable organic fluorides, such as fluoroacetic acid (Li et al., 2010; Stephanie et al., 2019) and 4-fluorothreonine (Wu et al., 2020b). However, the biosynthesis of organic fluorides by fluorinase is limited. On the one hand, fluoride ions have an inhibitory effect on the growth of *E. coli* (Last et al., 2016). On the other hand, fluorinase requires the expensive co-substrate S-adenosyl methionine (SAM) (Li et al., 2010; Feng et al., 2021; Kittila et al., 2022), and transmembrane transport of SAM requires the assistance of transporters (Schaffitzel et al., 1998). These all limit the catalytic efficiency of fluorinase. Therefore, there are few studies on the biosynthesis of organic fluorides.

**FIGURE 1:**
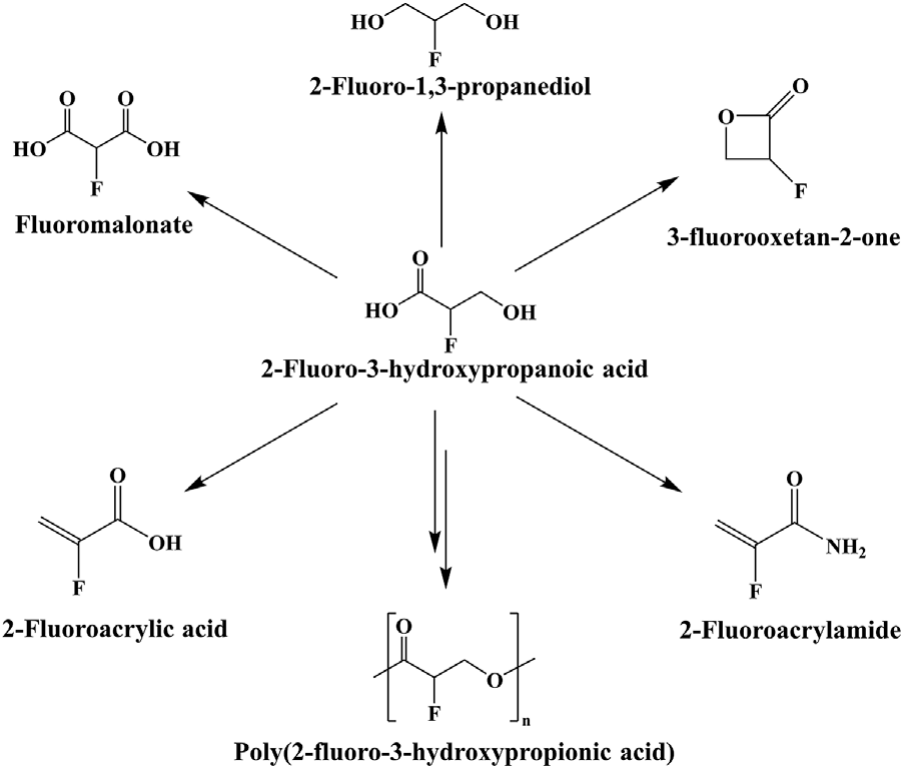
Potential products of 2-F-3-HP.

3-Hydroxypropionic acid (3-HP) is an important platform compound with a wide range of applications (Liu et al., 2016). It is easy to synthesize a variety of chemical products through different chemical reactions. For example, poly (3-hydroxypropionic acid) (P3HP) can be synthesized by 3-HP (Wang et al., 2013a; YongChang et al., 2014). Furthermore, malonate, 1,3-propanediol, acrylic acid, propiolactone and other products can be obtained through oxidation, reduction, dehydration, cyclization and other reactions, and the obtained products can be further used to synthesize products with higher added value (Valdehuesa et al., 2013). In theory, 2-fluoro-3-hydroxypropionic acid (2-F-3-HP) also produces many corresponding substances to expand the types of fluorine-containing organics, such as 2-fluoroacrylic acid (Figure 1). However, there is no biosynthetic pathway for 2-F-3-HP.

**SCHEME 1:**
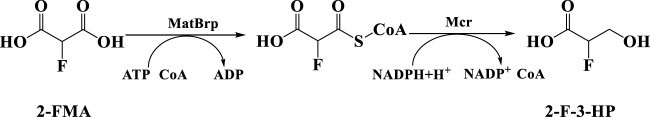
Synthesis of 2-F-3-HP designed in this study.

In this study, we constructed a four-gene, two-plasmid system for the biosynthesis of 2-F-3-HP using 2-fluoromalonic acid (2-FMA) as the initial fluorine source. 2-FMA is both relatively inexpensive and capable of being produced through enzymatic pathways while avoiding the acute organofluorine poisoning arising from the direct application of fluoroacetate to cells (Thuronyi et al., 2017). After whole-cell transformation, 2-F-3-HP (50.0 mg/L) was first synthesized by the engineered *E. coli* coexpressing methylmalonyl CoA synthase (MatBrp) from *Rhodopseudomonas palustris*, methylmalonyl CoA reductase (MCR) from *Thermophilic Filiculture* and malonate transmembrane protein (MadLM) from *Pseudomonas pf5* with 2-FMA as substrate (Scheme 1). The advantages of this method over chemical methods mainly included the use of green and cheap substrates, solvents and catalysts in mild and safe reaction conditions, and no toxic waste was generated. This method may be applicable to analogue of 2-F-3-HP, such as 2-fluoro-3-hydroxybutyric acid. Furthermore, according to the previous research in our lab, manipulating the expression levels of 3-hydroxybutyric acid (3-HB) and 3-HP pathways resulted in biosynthesis of block copolymers P3HB-b-P3HP with varied compositions, which improved properties of copolymers (Wang et al., 2013b). Theoretically, 2-F-3-HP and 2-fluoro-3-hydroxybutyric acid (2-F-3-HB), which has already been biosynthesized (Thuronyi et al., 2017), could also achieve similar results and possibly have better properties. In a word, the 2-F-3-HP synthesized in this study has broad application prospects.

## Materials and methods

### Materials

2-FMA and other chemicals were obtained from Aladdin (Shanghai, China). A DNA gel extraction kit, plasmid purification kit, Primer STAR Max and DNA marker were obtained from TAKARA (Japan). Protein markers and T4 DNA ligase were obtained from Thermo Fisher Scientific (United States). M9 Minimal Salts (M9 buffer) were obtained from Sangon Biotech (Shanghai, China). *E. coli* BL21 (DE3) competent cells were purchased from Vazyme (Nanjing, China).

### Construction of plasmids and strains

The *MatBrp* (WP_011155789.1), *Mcr* (AAS20429.1), *MadL* (AAY95003.2), *MadM* gene (WP_011063986.1) were synthesized by Beijing Genomics Institute (Beijing, China). The synthesized *MatBrp* was digested with *Bgl*II, and the synthesized Mcr was digested with *Bgl*II and *BamH*I. The digested *MatBrp* and *Mcr* were ligated into the pET28a vector, which was digested with *Nco*I, *EcoR*V and *Kpn*I. The MadLM gene was digested with *Bgl*II *and Xba*I, and the digested fragment was ligated into the pBAD, which was digested with the *EcoR*I, *Xba*I and *Hind*III. The constructed vector was transformed into *E. coli* BL21 (DE3).

### Enzyme activity analysis

*E. coli* cells BL21 (DE3) harboring recombinant plasmids were incubated at 37 °C for 12 h in 5 ml of LB medium (containing 100 μg/ml Amp and 34 μg/ml Cm, pH7.0). The grown cells (5 ml) were then transferred into 50 ml of LB medium (containing 100 μg/ml Amp and 34 μg/ml Cm, pH7.0) and cultivated at 37 °C in the thermostatic incubator (ZHICHENG ZWYR-D2403, China). When OD_600 nm_ reached 0.6, 0.5 mM isopropyl beta-d-1-thiogalactopyranoside (IPTG) and 0.02% arabinose were added. The cells were harvested by centrifugation (Himac CR21N, Japan) at 6,000 rpm for 5 min and washed with 100 mM Tris-HCl (containing 10 mM MgCl_2_, pH 7.8). After the cells (10 g of wet weight) were disrupted by a high-pressure cell cracker (Constant systems One shot 40KPSI, England), the cell debris was discarded, the supernatant (crude enzyme solution) was obtained by centrifugation (10000 rpm, 30 min, 4 °C). The standard assay was as followed: 1 mg/ml crude enzyme, 20 mM 2-FMA, 2 mM NADPH, 2 mM ATP, 1 mM CoA, 100 mM Tris-HCl buffer (10 mM MgCl_2_), pH 7.8, 30 °C, 200 rpm, 12 h. The reaction solution was detected by LC-MS.

### Whole-cell transformation

*E. coli* cells BL21 (DE3) harboring recombinant plasmids were incubated at 37 °C for 12 h in 5 ml of LB medium containing appropriate antibiotics. The grown cells (5 ml) were then transferred into 50 ml of LB medium containing appropriate antibiotics and cultivated at 37 °C in the thermostatic incubator. When OD_600 nm_ reached 0.6, 0.5 mM IPTG and 0.02% arabinose were added.

The cells were washed with M9 buffer (15.12 g/L Na_2_HPO_4_·12 H_2_O, 3 g/L KH_2_PO_4_, 0.5 g/L NaCl and 1 g/L NH_4_Cl, pH 7.0) to remove residual culture media and further resuspended in M9 buffer. The typical assay to directly measure reaction products was performed as follows: wet whole cells (OD_600 nm_ = 30), 4 mM 2-FMA, 8% glucose and 10 mM MgSO_4_. Reactions were performed in M9 buffer (pH 7.0) at 30°C with persistent stirring at 200 rpm. After centrifugation, the supernatants were analyzed by HPLC.

### Analytical methods

LC-MS conditions: ultra-high pressure liquid chromatography-triple quadrupole mass spectrometer (Agilent 1290–6430, United States); Mobile phase: A: H_2_O (0.01% formic acid); B: acetonitrile; Column: Acclaim Organic Acid (Thermo 2.1 × 150, United States); column temperature: 25°C; velocity of flow: 0.2 ml/min; linear gradient: 0% B, 0–4 min; 0%–30% B, 4–20 min; 30%-80%B, 20–22 min; 80% B, 22–26 min; 80%–0% B, 26–27 min; 0%, 27–32 min. Injection volume: 1 µL. MS conditions: Source: ESI; Scan type: negative, full scan; Scan range (m/z): 15–300; Fragmentor (V): 50; Cell Accelerator Valtage: 3; Capillary: 4.0 KV; Gas Temp: 350°C; Gas Flow: 9 L/min; Nebulizar: 35 psi; 0–2 min, LC to waste; 2–32 min, LC to MS.

HPLC conditions: High-performance liquid chromatography (Shimadzu, Japan); Mobile phase: 5 mM H_2_SO_4_; Column: HPX-87H (Bio-RAD, United States); column temperature: 50°C; velocity of flow: 0.5 ml/min; Injection volume: 5 µL.

### NMR identification

The fermentation broth is centrifuged for 5 min, the supernatant is collected, and the spin distillation concentrates to 1 ml. Take 500 μL of concentrate, add 10 μL of trifluoroacetic acid as the internal standard, add 100 μL D_2_O, transfer to a nuclear magnet tube to determine the chemical structure of 2-F-3 HP by fluorine spectrometry (^19^F-NMR).

## Results and discussions

### Design of synthetic pathway for 2-F-3-HP

In this study, 2-FMA was used as a substrate to biosynthesize 2-F-3-HP by the catalysis of MatBrp and MCR. The synthetic process of the 2-FMA was simple and mild. On the one hand, 2-FMA can be synthesized by microorganisms (Thuronyi et al., 2017). On the other hand, diethyl 2-FMA and lithium hydroxide monohydrate are used as raw materials to synthesize 2-FMA by chemical method, which is simple to operate and stable in process conditions (Darren et al., 2021). Furthermore, 2-FMA can be catalyzed by MatBrp to generate fluoromalonyl-CoA, therefore, theoretically, the conversion of 2-FMA to 2-F-HP could be achieved by co-expressing MatBrp and MCR.

The malonate transporter MadLM from *Pseudomonas* can transport 2-FMA into cells and improve the yield of fluoromalonyl-CoA (Schaffitzel et al., 1998; Thuronyi et al., 2017). Therefore, MadLM was co-expressed with MatBrp and MCR to verify the effect of MadLM on the yield of 2-F-3-HP. All strains and plasmids used in this study listed in Table 1.

**TABLE 1:** Strains and plasmids used in this study.

No.	Strains and plasmids	Description	Source
Strain 0	*E.coli* BL21 (DE3)	F-, ompT, hsdS (rBB-mB-), gal, dcm (DE3)	Addgene
Strain 1	BL21 (DE3)/pACYCDuet1/pBAD	BL21 (DE3) carrying pACYCDuet1 and pBAD	This study
Strain 2	BL21 (DE3)/pACYCDuet1-*MatBrp*-*Mcr*/pBAD-*madLM*	BL21 (DE3) carrying pACYCDuet1-*MatBrp*-*Mcr* and pBAD-*madLM*	This study
P001	pACYCDuet1	T7 promoter, lacIq, pBR322 ori, Cmr	Addgene
P002	pBAD	araBAD promoter, araCq, pBR322 ori, Ampr	Addgene
P003	pACYCDuet1-*MatBrp*-*Mcr*	T7 promoter, *MatBrp* and *Mcr*, lacIq, pBR322 ori, Cmr	This study
P004	pBAD-*madLM*	araBAD promoter, *madL* and *malM*, araCq, pBR322 ori, Ampr	This study

### Expression levels of MatBrp and MCR

**FIGURE 2:**
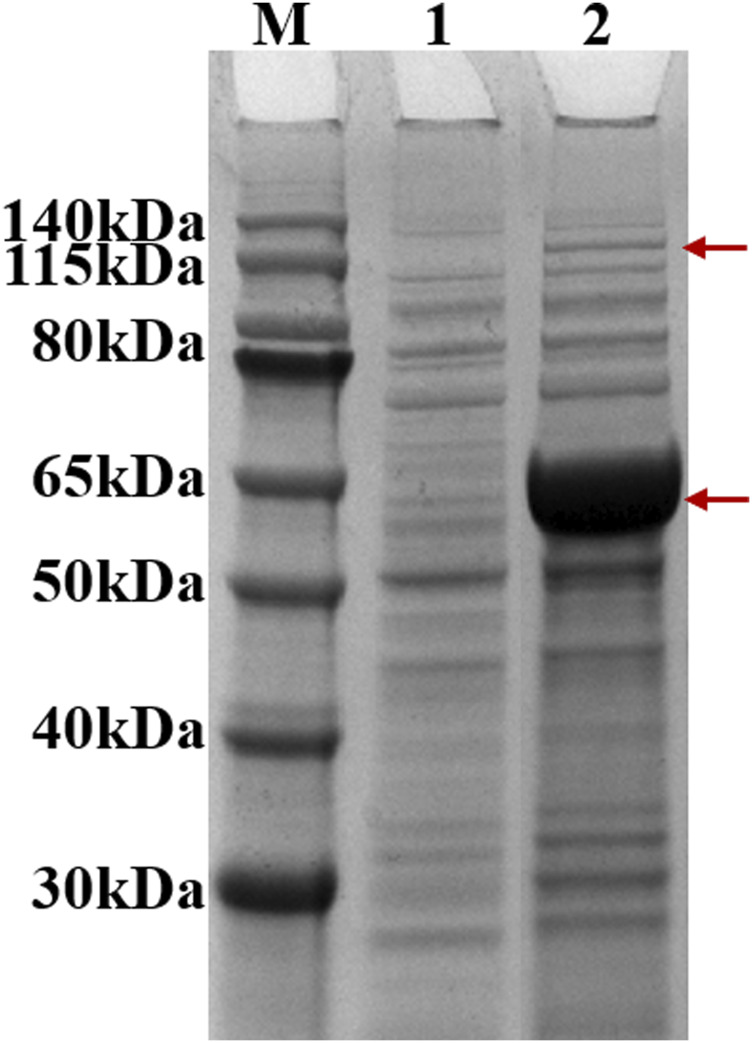
SDS-PAGE of crude enzymes. M: Marker; 1: strain 1 (BL21 (DE3)/pACYCDute1/pBAD); 2: strain 2 (BL21 (DE3)/pACYC-*matBrp-Mcr*/pBAD-*MadLM*).

After induction, recombinant strain 2 (Bl21 (DE3)/pACYCDuet1-*MatBrp*-*Mcr*/pBAD-*madLM*) was collected, and soluble expression levels of MatBrp and MCR were detected by SDS-PAGE. Compared with the control strain 1 (BL21 (DE3)/pACYCDute1/pBAD), MatBrp (55 kDa) and MCR (135 kDa) were expressed in the recombinant strain 2 (Figure 2). The soluble expression of MatBrp is significantly higher than that of MCR, which may be that MatBrp has a small protein molecular weight and is easier to fold correctly.

### Enzyme activity analysis

**FIGURE 3:**
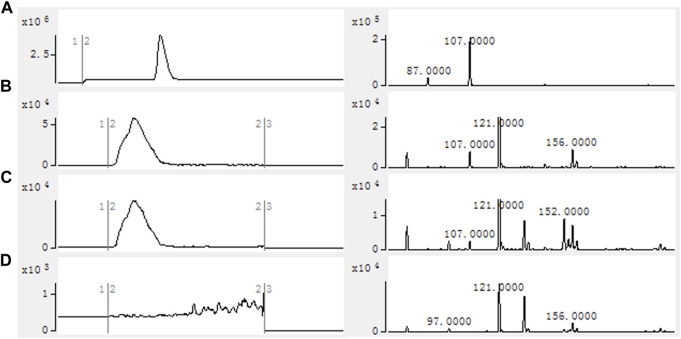
HPLC-MS results of *in vitro* catalytic reaction of crude enzyme. **(A)** 2-F-3-HP standard; **(B)** Strain 1 containing 2-F-3-HP standard; **(C)** Strain 2; **(D)** Strain 1. Reactions were performed in 100 mM Tris-HCl buffer (pH 7.8) containing 1 mg/ml crude enzyme, 20 mM 2-FMA, 2 mM NADPH, 2 mM ATP, 1 mM CoA, 10 mM MgCl_2_ at 30°C with 200 rpm shaking for 12 h. Strain 1: BL21 (DE3)/pACYCDuet1/pBAD; Strain 2: BL21 (DE3)/pACYCDuet1-*MatBrp*-*Mcr*/pBAD-*madLM*.

Using the crude enzyme of the recombinant strain as the catalyst, and NADPH, ATP and coenzyme A as the donors, the purpose was to verify that 2-FMA could be catalyzed by MatBrp and MCR to generate 2-F-3-HP. The formation of 2-F-3-HP was analyzed by HPLC-MS and the results are shown in Figure 3. Compared with strain 1, the reaction solution of strain 2 has a peak in about 2.50 min. In the strain 1 reaction solution containing 2-F-3-HP standard, the retention time of 2-F-3-HP was 2.5 min. Compared with strain 1, the reaction solution of strain 2 contains the characteristic peak EIC m/z = 107.0000. The results showed that MCR and MatBrp had the activity of catalyzing the formation of 2-F-3-HP. The retention time of the 2-F-3-HP standard is 3.0 min, which may be the complex environment of the crude enzyme system affecting the retention time of 2-F-3-HP, resulting in that the retention time of 2-F-3-HP in the sample was 2.5 min.

**FIGURE 4:**
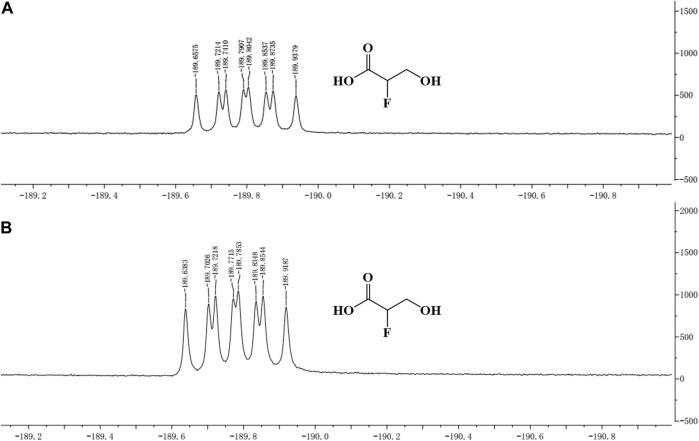
^19^F-NMR of 2-F-3-HP standard and sample. **(A)** ^19^F-NMR of the 2-F-3-HP standard; **(B)** ^19^F-NMR of the sample.

The concentrated reaction mixture was analyzed by ^19^F-NMR, and all fluorometabolites present in the samples were scanned. The same peak (similar chemical shift) as that of the standard was detected in the sample, which indicated that 2-F-3-HP was generated in the sample (Figure 4).

### Whole-cell biocatalytic synthesis of 2-F-3-HP

To assess the ability of strain 2 to produce the product, the titer of 2-F-3-HP was detected after whole-cell biocatalytic synthesis. Compared with strain 1, the concentration of 2-F-3-HP in strain 2 was 50.0 mg/L by HPLC detection (Table 2).

**TABLE 2:** The titer of 2-F-3-HP by whole-cell transformation.

Strain	Concentration (mg/L)
Strain 1	0
Strain 2	50.0

Reactions were performed in M9 buffer (pH 7.0) containing 4 mM 2-FMA, 8% glucose, 10 mM MgSO_4_, wet cells, OD_600 nm_ = 30 at 30°C with 200 rpm shaking for 24 h. Strain 1: BL21 (DE3)/pACYCDuet1/pBAD; Strain 2: BL21 (DE3)/pACYCDuet1-*MatBrp*-*Mcr*/pBAD-*madLM*.

The concentration of the product is very low, which may be the low activity of the MatBrp and MCR for non-natural substrate, or the toxicity of the 2-FMA to the cells. In the future, two key enzymes can be rationally designed to improve the enzymatic activity for the corresponding substrate.

## Conclusion

In this study, 2-F-3-HP was first synthesized by the engineered *E. coli* coexpressing MatBrp, MCR and MadLM with 2-FMA as substate. After whole-cell transformation, the 50.0 mg/L 2-F-3-HP was produced. 2-F-3-HP can be used as the substrate to synthesize other fluorides, such as poly (2-fluoro-3-hydroxypropionic acid) (FP3HP) or poly (2-fluoro-3-hydroxypropionic acid)-block-poly (2-fluoro-3-hydroxybutyric acid) (FP3HB-b-FP3HP), which is expected to obtain fluorine-containing materials with better properties.

## Data Availability

The original contributions presented in the study are included in the article/Supplementary Material, further inquiries can be directed to the corresponding author.

## Abbreviations

2-F-3-HP, 2-fluoro-3-hydroxypropionic acid; 2-FMA, 2-fluoromalonic acid; MatBrp, methylmalonyl CoA synthase; MCR, methylmalonyl CoA reductase; MadLM, malonate transmembrane protein; PHA, poly(hydroxyalkanoate); SAM, S-adenosyl methionine; 3-HP, 3-hydroxypropionic acid; 3-HB, 3-hydroxybutyric acid; 2-F-3-HB, 2-fluoro-3-hydroxybutyric acid; IPTG, isopropyl beta-d-1-thiogalactopyranoside; P3HP, poly (3-hydroxypropionic acid); P3HB, poly (3-hydroxybutyric acid); FP3HP, poly (2-fluoro-3-hydroxypropionic acid); FP3HB, poly (2-fluoro-3-hydroxybutyric acid); Amp, ampicillin; Cm, chloromycetin.
